# Supplementation Effect of Oleuropein Extract Combined with Betaine, Magnesium, and Vitamin E on Pigs’ Performance and Meat Quality Characteristics

**DOI:** 10.3390/ani11020443

**Published:** 2021-02-08

**Authors:** Ana I. Rey, Patricia Puig, Paul William Cardozo, Teresa Hechavarría

**Affiliations:** 1 Departamento Producción Animal, Facultad de Veterinaria, Universidad Complutense de Madrid, Avda. Puerta de Hierro s/n., 28040 Madrid, Spain; 2 Andres Pintaluba, S.A. Polígono Industrial Agro-Reus Prudenci Bertrana, 5, 43206 Reus, Spain; ppuig@pintaluba.com (P.P.); pcardozo@pintaluba.com (P.W.C.); thechavarria@pintaluba.com (T.H.)

**Keywords:** betaine, magnesium, oleuropein, vitamin E, meat quality, pork

## Abstract

**Simple Summary:**

Oleuropein, betaine, magnesium, and vitamin E show antioxidant and/or metabolic effects on the organism that are reflected, in many cases, in performances and meat quality. This study evaluated whether the combination of these nutrients at two doses manifest different effects on the final product. Both combinations were enough to improve the oxidative status of pigs, although performances were not affected. However, the higher doses increased n-6 and n-3 PUFA in the triglycerides and free fatty acid fractions that resulted in meat that was more susceptible to oxidation.

**Abstract:**

This study evaluates the effect of the dietary combination of oleuropein extract (1200 mg/kg) and betaine (1000 mg/kg), magnesium oxide (600 mg/kg), and α–tocopheryl acetate (400 mg/kg), or a half-dose of these compounds, on pigs’ performance, oxidative status, and meat quality characteristics (drip loss, TBARS, and texture and fatty acid profile of intramuscular fat). Sixty-six barrows and females were slaughtered at 120 kg of BW. Performance and carcass yield were not changed by treatments. The high-dose mixture resulted in higher serum ferric reducing/antioxidant power (*p* = 0.0026), lower glucose (*p* = 0.03) and a tendency to have lower serum TBARS (*p* = 0.07) when compared to control. Percentage of drip loss, moisture content, intramuscular fat, or texture parameters were not modified by dietary treatments. Pigs supplemented with the high-dose mixture had higher PUFA (*p* = 0.0001), n-6 (*p* = 0.0001), n-3 (*p* = 0.0095) and lower MUFA (*p* = 0.0064) in the neutral lipid fraction of intramuscular fat. Free PUFA, mainly n-3 fatty acids (*p* = 0.0009), were also higher in the meat of pigs fed the high-dose mixture compared with the others. A higher mobilization (neutral to free fatty acids hydrolysis) of n-3 and MUFA fatty acids in the muscle from pigs fed the high-dose mixture was observed. However, dietary mixture supplementation tended to increase MUFA (*p* = 0.056) and decrease the total PUFA (*p* = 0.0074) proportions in muscle polar lipids. This specific fatty acid composition of meat from pigs supplemented with the high-dose mixture could be responsible for the higher meat lipid oxidation observed in this group when compared to the other groups. Consequently, the low-dose mixture would be more adequate for maintaining the oxidative status of pigs and, meat lipid stability.

## 1. Introduction

Health status and wellness of animals in vivo has been related to the meat quality of final products [1,2]. Consequently, many dietary interventions have been investigated recently in order to ensure the increasing demand of high nutritional value and high-quality meat [3].

Oleuropein is one of the most abundant polyphenol compounds present in the olive leaves (*Olea europaea*) but it has also isolated from other plant species and fruits [4]. Recent studies show the positive effects of olive by-products, not only thanks to their antioxidant properties [4], but also due to their capacity to modify nutrient metabolism, with changes in glucose uptake, lipid, and amino acid profiles [5] that may affect meat quality [6,7]. Thus, oleuropein has shown capacity to catch free radicals and metal ions and it has been related with an increase in the glutathione route, cystine and monounsaturated blood levels, as well as reduced cortisol under stressful conditions [5] and other interesting pharmacological and anti-inflammatory effects [4]. In addition, the combination of olive extracts such as oleuropein has been reported to have synergistic effects in vivo with other antioxidants such as vitamin E [5,6]. Vitamin E is one of the most studied compounds in the literature because of its effects in vivo [5,8] and post-mortem [9], explained by its incorporation into biological membranes and consequent accumulation in different tissues. Vitamin E has been shown to be effective in improving colour and lipid stability, muscle proteolysis [10] or in reducing drip loss. However, their presence or function can be modified by the interaction with other compounds [9,11,12].

Betaine is the most effective methyl group donor [13] involved in energy and protein metabolism [14]. It is commonly added to diets because its supplementation increases energy value [13] and performance [15]. Dietary betaine supplementation has also been reported to modify some meat quality characteristics [16] and could have interesting effects in terms of controlling stress [14]. Recently, it has been found that the regulating fatty acid metabolism effect of betaine is associated with the up-regulation of genes involved in fatty-acid transportation and fatty-acid oxidation [17], which may modify the fatty acid profile. Moreover, the combination of dietary betaine and vitamin E has been reported to reduce meat TBARS and improve the oxidative status to a greater extent than betaine alone [18].

Another additive that participates in fat and glucose metabolism is magnesium. In this way, magnesium is involved in many enzymatic reactions in the organism and is essential for oxidation-reduction reactions and phosphorylation processes in charge of high-energy compounds [19]. Levels of 0.05–0.06% are recommended to fulfil requirements [20]. Magnesium has also been reported to improve meat quality, mainly by reducing drip loss [3], and it modifies the fatty acid profile in combination with selenium [21]. Hence, its in vivo effects on dyslipidaemia are explained by lipoprotein lipase and desaturase stimulation [22].

These compounds show antioxidant and/or metabolic effects on the organism that are reflected, in many cases, in meat quality. However, to our knowledge, the combination of these nutrients have not been evaluated together. Consequently, the aim of the present investigation was to study the effect of the dietary mixture at a higher dose of oleuropein extract (1200 mg/kg) with betaine (1000 mg/kg), magnesium oxide (600 mg/kg) and α–tocopheryl acetate (400 mg/kg), or a half-dose of these compounds (600 mg of oleuropein extract/kg; with 500 mg betaine/kg, 300 mg magnesium oxide/kg and 200 mg α–tocopheryl acetate/kg), on pigs’ performance and meat quality characteristics (drip loss, TBARS, colour changes and fatty acid profile of intramuscular fat).

## 2. Materials and Methods

The experimental protocols used in this study were approved by the research committee of the Veterinary Faculty of Complutense University of Madrid and complied with Spanish policy for Animal Protection [23], which is in accordance with the European Union Directive 2010/63/UE [24] on the protection of animals used for research.

### 2.1. Animals, Experimental Diets and Sample Collection

One hundred and seventeen pigs (barrows and females) (Large white × Landrace) × Duroc with an average live weight of 86.97 ± 4.86 kg were used. All pigs were identified with an electronic chip and were housed in an environmentally controllable room with slatted plastic flooring and a mechanical ventilation system (Odena Farm, Barcelona, Spain). Each pen was equipped with a self-feeder and nipple drinker to allow ad libitum access to feed and water throughout the experimental period (34 days). Pigs were distributed in 12 pens (10 pigs per pen, except for 3 pens with 9 pigs each), and allotted into 3 dietary treatments in a randomized complete block design according to their sex (50% castrated males and 50% female). Basal diet (Control) was similar for the different dietary treatments (Table 1) with the exception of the experimental mixture of the additives that was added at 1200 mg/kg oleuropein extract (*Olea europaea* L. leaves), 400 mg/kg α-tocopheryl acetate, 600 mg/kg magnesium oxide, and 1000 mg/kg betaine for the high-dose diet (MIX-4) and half-dose of each compounds for the low-dose diet (MIX-2). The products were provided by Pintaluba S.A. (Reus, Spain) and feeds were formulated and manufactured by Bon Area Group (Corporación Alimentaria Guissona, Lleida, Spain). Pigs were fed the experimental diets until final averaged weight of 120.0 ± 2.0 kg at the age of 167 ± 5 days (34 days of experimental period).

One day before slaughter and after a fasting time of 10 h blood samples were collected in 10 mL vacuum tubes from the jugular vein (10 pigs per treatment) and immediately placed on ice. The vacuum tubes containing blood samples were then centrifuged at 600× *g* for 10 min at 4 °C to obtain serum (the supernatant) that was collected, transported on dry ice and kept in a freezer at −80 °C until analysis (within 3 weeks).

Then, pigs (*n* = 66) were sent to a commercial slaughterhouse (Mafrica, San Joan de Villatorrada, Barcelona, Spain) where they were stunned and slaughtered after a fasting time of approximately 18 h. Carcasses of 22 animals per treatment were automatically scanned before entering the pre-scald using AutoFom (ultrasounds) and gmScan (magnetic induction). Data were obtained on lean percentage, thickness of the back fat, thickness of the loin, ham weight, lean percentage of ham, and thickness of ham. Then, carcasses were eviscerated, split down and chilled at 4 °C.

Samples from the *Longissimus lumborum* muscle (*n* = 30; 10 samples per treatment) were taken at the level of the last rib and chop cuts of 2 cm thickness were placed in plastic boxes, transported on dry ice, and stored in vacuum-packed plastic bags at −20 °C until analysis.

### 2.2. Laboratory Analysis

#### 2.2.1. Biochemical Parameters and Antioxidant Capacity in Serum Samples

Glucose was determined using a kit according to manufacturer instructions (Glucose GOD-PAD kit, Materlab, Madrid, Spain).

The antioxidant capacity of serum samples was determined by the ferric reducing antioxidant power (FRAP) procedure [25]. The FRAP reagent (mixture of 300 mM acetate buffer with 10 mmol 2,4,6-tripyridyl-s-triazine in 40 mM HCl and 20 mM aqueous ferric chloride at 10:1:1 proportions) was prepared fresh. Sample extract (100-μL) was mixed with the working FRAP solution (3 mL) and the absorbance of the samples (593 nm) (ScanGo, ThermoFisher Scientific, Alcobendas, Spain) was taken after 0 and 4 min. Results were expressed as μM.

#### 2.2.2. Tocopherol Content of Serum and Muscle Samples

The α-tocopherol concentration in serum and muscle samples was quantified by direct extraction as described by Rey et al. [26]. Samples in presence of dibasic sodium phosphate buffer (0.054 M adjusted to pH 7.0 with HCl) and absolute ethanol were mixed (in serum) or homogenized (in muscle samples). Then, the tocopherol was extracted with repeated hexane addition by centrifugation and the upper layer was evaporated to dryness under N_2_ stream. Tocopherol was dissolved in ethanol and it was analysed (in duplicate) by reverse phase HPLC (HP 1100, equipped with a diode array detector) (Agilent Technologies, Waldbronn, Germany) as described elsewhere [26]. Quantification was carried out by means of a standard curve (R^2^ = 0.999) built using the pure compound (Sigma, Alcobendas, Madrid, Spain).

#### 2.2.3. Drip Loss in Muscle Samples

Drip loss was determined in a fresh chop of *Longissimus lumborum* muscle (10 g and approximately 1 cm^3^). After weighing samples were put inside of a mesh that was placed in a plastic bag under refrigerated conditions at 4 °C. To calculate drip loss, samples were weighed again after 72 h of storage and the difference between initial and final weights was used for calculation, which was expressed as percentage of the initial weight [10].

#### 2.2.4. TBARS of Serum and Muscle Samples

The thiobarbituric acid reactive substances (TBARS) were quantified in serum samples according to a spectrophotometric method as described elsewhere [27]. Deproteinized serum by perchloric acid addition were iron-induced to oxidation (1 mM FeSO_4_), mixed with thiobarbituric acid (1:2) and heated at 100 °C. After centrifugation (600× *g* for 10 min at 4 °C) absorbance was read at 532 nm (ScanGo, ThermoFisher Scientific, Alcobendas, Spain). Results were expressed as μmoles MDA (malondialdehyde)/L serum.

To evaluate the effects on lipid oxidation of meat, TBARS were measured in pork chops of *longissimus* muscle (approximately 40 g) on day 0, 4, and 7 of refrigerated (4 °C) display [8]. Chops were placed on polystyrene trays, over-wrapped with an oxygen permeable PVC wrap (6000–8000 mL O_2_/m^2^/24 h at STP) and kept at 4 °C under fluorescent light (616 lux). At different time intervals, samples in presence of perchloric acid were homogenized, filtered and mixed with thiobarbituric acid. Absorbance was measured spectrophotometrically at 532 nm (ScanGo, ThermoFisher Scientific, Alcobendas, Spain). TBARS were expressed as mg MDA/kg muscle. In both analysis TBARS concentrations were calculated using 1.56 × 105 M-1 × cm^−1^ as the molar absorption coefficient.

#### 2.2.5. Texture Parameters Measurement

Texture parameters (harness, adhesiveness, springiness, cohesiveness and gumminess) were measured in muscle cuts (four per sample) using a Texture Analyzer (TA.XT 2i/SMS Stable Microsystems Ltd., Surrey, UK). Measurements were calculated and expressed as follows: Hardness = maximum strength to achieve compression measured in Newtons (N), adhesiveness = area under the abscissa after the first compression expressed in Newtons × second (Nxs), springiness = height that sample recovers between the first and second compression measured in meters (m), cohesiveness = ratio of work under the curves, gumminess (N) calculated as hardness × cohesiveness, chewiness expressed in Joules (J) and calculated as harness × springiness × cohesiveness.

#### 2.2.6. Lipid Fractions and Fatty Acid Profile of Intramuscular Fat

Lyophilized muscle samples were weighed and homogenized in presence of dichloromethane-methanol (8:2), in a mixer mill (MM400, Retsch technology, Haan, Germany). Then, samples were centrifuged (8 min at 10,000 rpm) and the solvent was collected and evaporated under nitrogen stream [28]. The intramuscular lipid content was gravimetrically quantified. After fat extraction, lipids were fractionated in neutral lipids (NL), free fatty acids (FFA) and polar lipids (PL) [29]. Briefly, intramuscular fat (20 mg) was dissolved in hexane:chloroform:methanol (95:3:2) and this mixture was added to an aminopropyl minicolumn (Varian, Harbor City, CA, USA) (previously activated with 7.5 mL of hexane). NL were eluted with chloroform (5 mL), FFA were eluted with diethylether:acetic acid (98:2) and PL were eluted with 2.5 mL methanol:chloroform (6:1) and sodium acetate in methanol:chloroform (6:1). Lipid fractions were subjected to esterification [5,6]. The fatty acids methyl esters (FAMEs) were directly injected and separated in a gas chromatograph (HP 6890 Series GC System; Hewlett Packard, Avondale, PA, USA) equipped with flame ionization detector held at 250 °C and a capillary column (HP-Innowax Polyethylene Glycol, 30 m × 0.316 mm × 0.25 µm). Nitrogen was used as a carrier gas. After injection at 170 °C, the oven temperature was raised to 210 °C at a rate 3.5 °C/min, then to 250 °C at a rate of 7 °C·min^−1^ and held constant for 1 min. FAME peaks were identified by comparing their retention times with those of authentic standards (Sigma-Aldrich, Alcobendas, Spain) and quantified by g per 100 g quantified fatty acids.

#### 2.2.7. Statistical Analysis

Data were analysed using the general linear model (GLM) procedure contained in SAS (version 9; SAS Inst. Inc., Cary, NC, USA). Data were presented as the mean and the standard error (s.e.) of each group together with significance levels (*p* value) of the main effects and interactions. Duncan’s test was used to separate treatment means. Differences between means were considered statistically significant at *p* < 0.05.

## 3. Results

The supplementation of two treatments in fattening pigs diet did not modify (*p* > 0.05) the pigs’ performance (body weight: BW or average daily gain: ADWG, average daily intake: ADFI and feed conversion ratio: FCR) (Table 2). Carcass yield (carcass lean %, lean % of ham and loin, carcass fat thickness, and weight or percentage of ham, shoulder, loin, or belly) was changed by neither dietary supplementation of the two additive-mixture (*p* > 0.05).

Glucose was reduced with the high-dose mixture (*p* = 0.03) (Table 3), whereas the use of the lower doses did not affect (*p* > 0.05) glucose levels when compared to the control group. However, the use of the half-dose mixture increased the concentration of α-tocopherol in serum (*p* = 0.0001) when compared with the control group, but no differences were observed with the high-dose mixture. Moreover, supplemented groups resulted in higher serum FRAP (*p* = 0.0026) and a tendency to have lower serum TBARS (*p* = 0.07) when compared to the control.

The muscle composition of *longissimus lumborum* is presented in Table 3. Percentage of drip loss, moisture content and intramuscular fat were not modified by treatments (*p* > 0.05). On the contrary, α-tocopherol in the muscle increased with the supplementation dose (*p* = 0.0011), whereas TBARS production increased in the group supplemented with the high-dose mixture at day 7 of refrigerated storage when compared to the control (*p* = 0.033).

In terms of the texture parameters of muscle (Table 3), no changes were found with dietary supplementation in muscles’ hardness, adhesiveness, springiness, cohesiveness, gumminess and chewiness when compared to the control group (*p* > 0.05).

The intramuscular fatty acid profiles of neutral, free and polar fatty acids are presented in Table 4, Table 5 and Table 6, respectively. The high-dose mixture resulted in higher C18:2n-6 (*p* = 0.0001), C20:3n-6 (*p* = 0.0006), C20:4n-6 (*p* = 0.0001), C22:4n-6 (*p* = 0.0001), and C22:6n-3 (*p* = 0.0001) in the neutral lipid fraction, whereas C18:1n-9 (*p* = 0.0092) and C22:5n-3 (*p* = 0.0001) were lower in neutral lipids when compared with the other groups. The half-dose mixture did not differ in most of the fatty acids of the neutral lipid fraction compared with the control group, except for C20:5n-3 and C22:5n-3 that had a lower proportion compared with the control (*p* < 0.0001). These effects resulted in a higher proportion of PUFA (*p* = 0.0001), n-6 (*p* = 0.0001), n-3 (*p* = 0.0095), and n6/n3 (*p* = 0.0001) in the neutral fraction of the high-dose mixture supplemented pigs, whereas the proportion of total MUFA was lower (*p* = 0.0064). No changes between the control and half-dose mixture were detected in the main groups of fatty acids.

Changes were also observed in the free fatty acid fraction (Table 5). Pigs supplemented with the high-dose mixture had a higher proportion of C20:0 (*p* = 0.0001), C20:3n-6 (*p* = 0.0001), C22:4n-6 (*p* = 0.0034), C22:5n-3 (*p* = 0.0003), and C22:6n-3 (*p* = 0.0003) in the free fatty acid fraction compared with the other groups. Consequently, pigs that received the high-dose mixture had a higher proportion of n-6 (*p* = 0.033) and n-3 (*p* = 0.0009) free fatty acids when compared to the other groups, and tended to have higher free PUFA (*p* = 0.06) when compared to the control. Pigs given the half-dose mixture had a similar free fatty acid proportion to the control (*p* > 0.05).

The fatty acid composition of the polar lipid fraction is presented in Table 6. Pigs supplemented with the high-dose mixture had lower C16:1n-9 (*p* = 0.0001), C16:1n-7 (*p* = 0.0002), C17:0 (*p* = 0.0001), C17:1 (*p* = 0.0001), C18:2n-6 (*p* = 0.0021), C18:3n-3 (*p* = 0.043) and PUFA (*p* = 0.0074) and tended to have higher MUFA (*p* = 0.056) in the polar lipid fraction when compared to the control group. Pigs fed the half-dose mixture did not differ in SAT or MUFA polar lipid fractions with the aforementioned fatty acids, except for C18:2 n-6 (*p* = 0.0021) and PUFA proportions (*p* = 0.0074), which were lower compared with the control group and similar to those reported for the higher-dose mixture.

**Figure 1:**
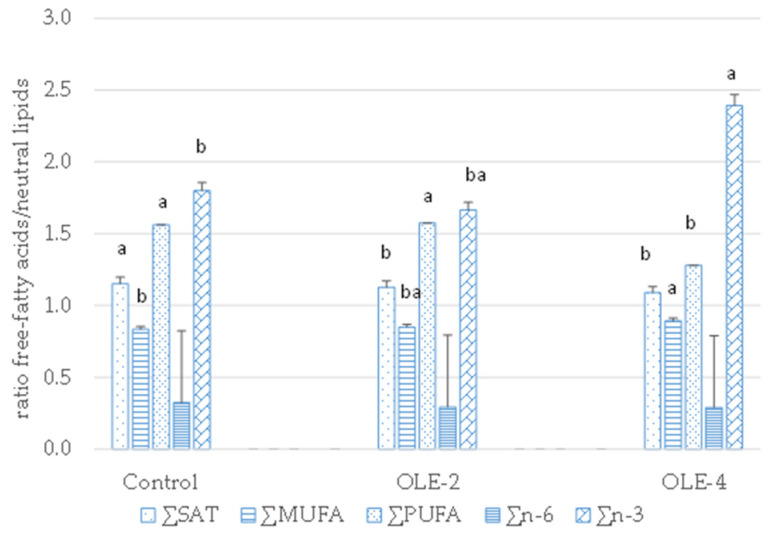
Relative fatty acid mobilization/hydrolysis (ratio free-fatty acids/neutral lipids) of intramuscular fat according to dietary oleuropein extract combined with betaine, magnesium and α-tocopheryl acetate (at 2 or 4 kg/Tn feed). Different letters (a, b) indicate *p* < 0.05.

The ratio free-fatty acids/neutral lipids as indicator of relative mobilization/hydrolysis is presented in Figure 1. Pigs supplemented with the high-dose mixture had higher n-3 and MUFA values (*p* < 0.05) and lower SAT and PUFA ratios. Pigs fed the half-dose mixture had intermediate values of n-3 and MUFA and did not differ in PUFA ratio when compared with the control group.

## 4. Discussion

Most studies have evaluated the effects of betaine, oleuropein, vitamin E, or magnesium on meat quality separately. This is the first study evaluating the combined effects of these nutrients. These compounds were chosen due to their positive effects as antioxidant agents, so they would be expected to have additive effects on pig’s meat quality. Two different combinations of doses (double and half dose) were therefore investigated in order to give the most recommended mixture’s dose.

In the present study, no differences in ADWG, ADFI, or FCR were observed in pigs supplemented with the experimental diets and a lack of effects on lean or fat thickness of carcass yield was observed. Similar results have been reported through the single administration of vitamin E and/or oleuropein extract [6,30]. Other feed additives, such as magnesium, have been reported to improve the performance at 0.3% (3000 mg/kg) of MgO on the diets of swine over 7 days [31]. Moreover, these authors also found decreased backfat thickness and improved carcass yield depending on the magnesium supplementation dose. However, the doses used in the present study were lower (300 and 600 mg/kg) than those used by Tarsitano et al. [31]. In terms of betaine supplementation, there are contradictory results; Fernandez-Figares et al. [32] observed no effects on ADWG and FCR in pigs (from 36 to 64 kg) using betaine at 0.12, 0.25, or 0.5% (1200, 2500, or 5000 mg/kg), but found fat concentration to be reduced by treatments. However, Siljander-Rasi et al. [33] found that the use of betaine at 250, 500, and 1000 mg/kg resulted in a linearly improvement of the ADWG and F/G ratio. In a meta-analysis carried out by Sales [34], betaine supplementation (from 1000 to 5000 mg/kg) in growing-finishing pigs decreased carcass fat, whereas no significant effect was found on ADWG. In the present study, the combination of these four compounds did not show additive effects on these parameters.

Concerning the effect of the half-dose or high-dose mixture on blood parameters as indicators of the glycemic muscle state that could modify meat characteristics, a decrease of serum glucose was observed with the high-dose mixture supplementation, which might be explained by the described hypoglycemic effect of oleuropein extract [35] or its combination with other compounds such as magnesium or betaine. The lack of effects of the half-dose mixture on diminishing glucose levels was also observed in other works when lower doses of oleuropein (192 mg/kg) were combined with vitamin E (100 mg/kg) and selenium (0.26 mg/kg) [5], and this was attributed to the possible contrary effects of these compounds on glucose concentrations. A lower accumulation of vitamin E has therefore been associated with lower blood glucose [36]. Meanwhile, it has been reported that lower serum levels of magnesium are related to increased glycaemia in patients with diabetes [37,38]. However, betaine supplementation has shown effects on decreasing glucose at lower doses (15 g/d vs. 30 g/d) [39] with possible effects depending on the physiological state [40]. The half-dose mixture of oleuropein, vitamin E, magnesium, and betaine did not, therefore, modify glucose when compared to the control and would need a high-dose mixture to reduce this blood parameter.

The accumulation of α-tocopherol in serum and muscle was related with the supplementation dose and higher numbers were detected in those pigs that received more of this vitamin in feed. These differences in serum α-tocopherol could result in a favourable oxidative status in mixture-supplemented animals, however, a dose effect was not observed and half-dose and high-dose supplemented pigs had similar serum α-tocopherol and total antioxidant power. Other studies have shown the relationship between increased serum α-tocopherol concentration and antioxidant power [8,27], which is explained by the potent antioxidant effect of this vitamin [8]. Moreover, lower doses of oleuropein extract (192 mg/kg) and a similar supplementation time than those used in the present study have shown antioxidant effects in vivo, explained by their contribution of the glutathione route [5] as one of the most interesting antioxidant enzyme complexes in the organism. Other nutrients such as magnesium have been negatively associated with oxidative stress and increased TBARS production [41], and it has been suggested that, in humans, magnesium may modulate antioxidant defenses in the organism [42]. However, betaine supplementation has shown certain prooxidant effects [43], whereas other studies show antioxidant [44] or prooxidant effects depending on the dose administered [39]. Finally, the combination of all these dietary nutrients together in the present research resulted in a favourable blood oxidative status of supplemented animals that was surprisingly not reflected in the oxidative status of meat, since the high-dose mixture supplemented pigs had a higher content of TBARS production at day 7 of refrigerated storage when compared to the control. This result was not expected taking into consideration that vitamin E concentration was higher in those pigs supplemented with the high-dose mixture, as this group also had the highest concentration of serum α-tocopherol and the relationship found between serum α-tocopherol and their accumulation in tissues [8,27]. Vitamin E is one of the most potent antioxidants in the organism that can be accumulated in muscle membranes, so a higher presence of this vitamin has been associated with lower TBARS production and higher lipid stability [9]. In addition, the use of oleuropein extract has been reported to delay lipid oxidation in meat from birds [30] and pork [6], and similar results have been found in pork through the use of olive leaves that may contain not only oleuropein but also other antioxidant components [45]. Lower TBARS values have also been observed in meat from pigs given a short-term feeding of magnesium supplements [46], although supplementation doses were much higher (1500 mg/kg) than those used in the present research. In addition, the use of betaine (1000 mg/kg) alone [47] or in combination with 200 IU/kg of vitamin E improved the TBARS production of meat [18]. It has been reported that betaine participates in the methionine cycle through the remethylation of homocysteine to methionine, which is a precursor of cysteine, taurine, and glutathione, which have been reported to have antioxidant effects [5,18]. Despite the antioxidant effects of the different nutrients used in the present study, results indicate an imbalance in oxidative status between antioxidant–prooxidant components in the muscle that might be explained by differences in muscle composition not only depending on vitamin E accumulation as reported later. Moreover, the accumulation of these antioxidants in meat could be affected by the supplementation dose; however, in the present study their concentrations were not quantified in muscle with the exception of vitamin E. Hence, previous studies reported that bioavailability and tissue utilization of lipophilic antioxidants maybe affected by other antioxidant compounds of hydrophilic characteristics [48,49]. Since there is not previous studies in which these four compounds were combined it is difficult to assess the single supplement responsible of the specific effect or the interaction with other constituents. So further investigations would be needed to dilucidate this fact.

Concerning other meat quality characteristics, no differences were observed in the present study on the combination of different nutrients in drip loss or texture parameters. Pigs supplemented with the high-dose or low-dose mixture only showed a tendency to have higher water-holding capacity but no significant changes were found. The use of olive by-products at 5 or 10% [45], or their combination with vitamin E [6], has been reported to have positive effects on drip loss. Dietary supplementation with MgO at doses of 0.3% (3000 mg/kg) [31] or higher (3.6 g/pig/day) [50] has also been effective in reducing the drip loss of pork, however, other authors using doses of 5 g/pig/day in the form of magnesium oxide did not find clear effects [46]. On the other hand, betaine has also shown positive effects on the water retention of meat [47,51]. Doses of magnesium and betaine in the present study were lower than those in which positive effects were found. However, vitamin E and oleuropein were higher than those in which positive effects on these parameters were reported (100 mg/kg of vitamin E and 192 mg/kg of oleuropein). Since water retention is related with proteolysis and texture of muscle [10], these measurements were also quantified. As with drip loss, no significant changes were found by treatments. Other authors found a lack of effect in meat texture through the single dietary administration of magnesium oxide [46] or betaine [47,51], even though it affected drip loss. There is no previous information on the combined effect of oleuropein extract, vitamin E, MgO, and betaine, but in terms of the present results, the combination of these nutrients did not show additive effects on water retention or texture parameters.

The fatty acid profile of meat was one of the characteristics most affected by the combination of the different nutrients at different doses. It has been reported that it is mainly oleuropein, betaine and magnesium that have effects on lipid metabolism through different mechanisms that result in faster lipid utilization. In the present research, pigs supplemented with the high-dose mixture had higher PUFA, n-6, n-3 and lower MUFA in the neutral lipid fraction of intramuscular fat. Free PUFA were also higher in the meat from high-dose mixture supplemented animals when compared to the others. In a previous study, doses of oleuropein around 200 mg/kg decreased total PUFA proportion and increased relative mobilization index (free-fatty acids/neutral lipids) in blood [5], whereas the proportion of PUFA free fatty acid of muscle decreased [6]. This was attributed to a faster glucose uptake and lipolysis initiation. However, according to the results of the present study, the combination of oleuropein at 1200 or 2400 mg/kg with other three compounds would result in a different fatty acid profile. The fatty acid profile could also be affected by betaine supplementation, although probably to a lesser extent due to the supplementation dose. According to Xu et al. [52], the efficiency of betaine supplementation on decreasing unsaturated fatty acids is reduced at levels rising above 0.08% (800 mg/kg), which is lower than the high-dose supplementation of the present study 1000 mg/kg. In other studies, it has been reported that betaine at doses of 0.2% (2000 mg/kg) [16] or higher decreased unsaturated fatty acids and increased saturated fatty acids in pork. Similar results were observed by Yang et al. [53], who reported lower PUFA in the loin of pigs supplemented with 2000 mg/kg of betaine, but did not find changes in the PUFA proportion of meat from pigs fed 4000 or 6000 mg/kg. Li et al. [17] found that betaine at 1250 or 2500 mg/kg (0.1–0.2%) increased muscle free fatty acids (similarly to what was observed in the present study for the high-dose mixture). This was attributed to differences in the balance of fatty acid uptake and oxidation, since betaine promotes fatty acid uptake, increasing the expression of transporters and enhancing fatty acid oxidation through AMPK activation. Moreover, betaine indirectly stimulates the synthesis of carnitine necessary for the transport of long-chain fatty acids to mitochondria, where they are oxidized [54]. So according to the effects reported for oleuropein or betaine supplementation, PUFA proportions should have been depleted in pigs supplemented with the mixture. A higher uptake of n-6 and n-3 PUFA in the triglycerides fraction of intramuscular fat was observed, however, a higher hydrolysis of n-3 fatty acids was only observed in the muscle from pigs fed a high-dose mixture. It is possible that their effects could be counteracted by magnesium supplementation. Therefore, the higher proportion of PUFA in meat from the high-dose mixture might be attributed in part to the effects of this mineral on the fatty acid profile, as reported previously [21]. The authors using 300 mg/kg of magnesium oxide reported higher proportions of PUFA and n-6 and n-3 fatty acids in pig muscle that was attributed to their effects on some desaturase enzymes. Hence, magnesium has been reported to be a cofactor for Δ5 and Δ6-desaturases [55]. However, because in the present study four compounds were used in the dietary mixtures it is not possible to quantify the specific effect of each component on fatty acid profile.

A higher mobilization was also observed in MUFA in accordance with previous findings on oleuropein supplementation [5], but this group of fatty acids was also found in the present study at higher proportions in polar lipids of muscle membranes of pigs fed the high-dose mixture, which would indicate that its utilization for energy supply was not as important as other unsaturated fatty acids [56]. In addition, antioxidants have been reported to protect Δ9 desaturase enzymes [9,57] mainly in the lipid fraction [9].

Despite the high potential of lipid mobilization of oleuropein and betaine, muscle from pigs supplemented with the high-dose mixture had higher PUFA in the triglyceride and free fatty acid fractions. This specific fatty acid composition of meat from pigs supplemented with the high-dose mixture could be responsible for the highest lipid oxidation observed in this group when compared to the control. Phospholipid fraction has been considered to be the main agent responsible for lipid oxidation initiation [58], since PUFA are mainly located in lipid membranes. However, other authors have found a direct relationship between muscle free fatty acids and TBARS production [59] and an inverse or direct correlation between serum free n-3 and free MUFA fatty acids and muscle lipid stability [6].

## 5. Conclusions

In conclusion, dietary supplementation of oleuropein (600 mg/kg), vitamin E (400 mg/kg), magnesium (300 mg/kg), and betaine (500 mg/kg), described as a half-dose mixture, was enough to improve the oxidative status and maintain lipid stability of the pig’s meat. The high-dose mixture reduced blood glucose levels and increased n-6 and n-3 PUFA in the triglycerides and free fatty acid fractions that resulted in meat that was more susceptible to oxidation. Consequently, the lower dose combination would be more adequate for maintaining the lipid stability of meat. Further studies would be needed to dilucidate the specific mode of action in the mixture system or possible interactions between compounds.

## Figures and Tables

**Table 1:** Major nutrients of the experimental diets.

Nutrients	%
Protein	15.08
Crude fat	6.69
Crude fiber	5.18
Neutral detergent fiber (NDF)	14.87
Starch	41.86
Sugar	3.12
Ash	4.91
Calcium	0.60
Total phosphorous	0.55
Net energy (Mcal/kg)	2.44

Ingredients (%): barley 32.98; wheat 20.00; corn 15.00; rice flour 10.00; soya 47 8.95; sunflower 30/8.5 7.5; palm oil 1.76; fat 1.00; calcium carbonate 0.93; L-lysine 0.51; salt 0.45; bi-calcium phosphate 0.37; premix 0.20; L-threonine 0.15; DL-methionine 0.09; carbohydrase enzyme 0.05; phytase 0.02; L-tryptophane. Premix (content per kilogram): 13.008 UI of vitamin A; 1.001 UI of vitamin D3; 150 ppm of vitamin E; 5 ppm of vitamin K; 3 ppm of vitamin B1; 8 ppm of vitamin B_2_; 6 ppm of vitamin B_6_; 0.05 ppm of vitamin B_12_; 1.5 ppm of folic acid; 55 ppm of nicotinic acid; 30 ppm of pantothenic acid; 0.3 ppm of Biotin; 25 µg of Vitamin 25-OH-D3; 74 ppm of Copper; 1.01 ppm of Iodine; 33 ppm of Manganese; 0.15 ppm of Selenium; 63 ppm of Zinc.

**Table 2:** Effect of dietary oleuropein extract combined with betaine, magnesium and α-tocopheryl acetate (at 2 or 4 kg/Tn feed) on pigs performances and carcass yield.

Pigs’ Performances	Treatments ^1^				
	Control	MIX-2	MIX-4	SEM ^2^	*p* Value
Initial body weight, kg	86.44	87.08	87.58	2.671	0.9567
Body weight at day 34, kg	119.52	120.76	119.87	2.075	0.9128
ADWG total, g/d	0.99	0.99	0.96	0.032	0.8054
ADFI total, g/d	3.05	3.02	3.01	0.098	0.9388
FCR total, g/g	3.09	3.05	3.13	0.158	0.9312
Carcass Yield					
Lean, %	51.79	52.26	53.27	1.059	0.6027
Fat thickness at 3–4 rib, mm	25.48	25.00	24.02	1.127	0.6509
Lean thickness at 3–4 rib, mm	55.99	55.65	56.60	0.934	0.7659
Fat thickness of ham, mm	33.61	33.58	32.39	1.346	0.7639
Lean percentage of ham	65.64	66.14	66.48	0.906	0.8038
Lean percentage of loin	57.70	57.96	58.39	0.694	0.7732
Ham Weight, kg	11.72	11.67	11.69	0.100	0.9406
Belly Weight, kg	4.44	4.46	4.45	0.056	0.9452
Shoulder Weight, kg	6.60	6.64	6.65	0.061	0.8168
Loin Weight, kg	8.19	8.14	8.14	0.086	0.8893
Ham Lean, %	68.09	68.22	68.52	0.474	0.8100
Belly Lean, %	54.26	54.55	54.95	0.680	0.7705
Shoulder Lean, %	65.28	65.39	65.64	0.420	0.8233
Loin Lean, %	57.70	57.96	58.39	0.694	0.7732

^1^ Control = Non additives; MIX-2 = 2 kg/Tn of oleuropein extract (600 mg/kg) with betaine (500 mg/kg), magnesium oxide (300 mg/kg) and α–tocopheryl acetate (200 mg/kg); MIX-4 = 4 kg/Tn of oleuropein extract (1200 mg/kg) with betaine (1000 mg/kg), magnesium oxide (600 mg/kg) and α–tocopheryl acetate (400 mg/kg); ^2^ SEM: standard error of the mean; *n* = 22.

**Table 3:** Effect of dietary oleuropein extract combination with betaine, magnesium and α-tocopheryl acetate (at 2 or 4 kg/Tn feed) on serum parameters and muscle composition, stability, and texture.

	Treatments ^1^			SEM ^2^	*p* Value
	Control	MIX-2	MIX-4		
Serum parameters					
Glucose, mg/100 mL	90.40 ^a^	92.57 ^a^	78.56 ^b^	3.692	0.0308
α-tocopherol, µg/mL	4.95 ^b^	7.87 ^a^	9.04 ^a^	0.497	0.0001
TBARS, mmoles/L	0.0043 ^a^	0.0041 ^ab^	0.0035 ^b^	0.000	0.0704
FRAP, µM	94.70 ^b^	135.38 ^a^	139.98 ^a^	9.263	0.0026
Muscle composition					
Drip loss, %	9.673	7.754	8.374	0.523	0.1479
Moisture, %	73.713	74.040	74.306	0.226	0.1966
Intramuscular fat, %	3.231	3.004	2.724	0.274	0.4353
α-tocopherol, ug/g	2.333 ^b^	3.004 ^ab^	3.373 ^a^	0.177	0.0011
Meat quality traits					
Drip loss, %	9.673	7.754	8.374	0.523	0.1479
TBARS (mg MDA/kg)					
Day 0	0.116	0.126	0.126	0.010	0.6070
Day 4	0.269	0.289	0.307	0.031	0.5188
Day 7	0.228 ^b^	0.307 ^ab^	0.343 ^a^	0.039	0.0330
Texture parameters					
Hardness, N	50.828	42.258	44.198	4.005	0.3000
Adhesiveness, Nxs	−0.452	−0.468	−0.480	0.031	0.8166
Springiness, m	0.001	0.001	0.001	0.000	0.4945
Cohesiveness	0.421	0.413	0.417	0.013	0.9142
Gumminess, N	21.340	17.287	18.297	1.650	0.2135
Chewiness, J	0.023	0.027	0.018	0.006	0.5423

^1^ Control = Non additives; MIX-2 = 2 kg/Tn of oleuropein extract (600 mg/kg) with betaine (500 mg/kg), magnesium oxide (300 mg/kg) and α–tocopheryl acetate (200 mg/kg); MIX-4 = 4 kg/Tn of oleuropein extract (1200 mg/kg) with betaine (1000 mg/kg), magnesium oxide (600 mg/kg) and α–tocopheryl acetate (400 mg/kg); ^2^ SEM: standard error of the mean; *n* = 10. Different letters (^a,b^) indicate *p* < 0.05.

**Table 4:** Effect of dietary oleuropein extract combination with betaine, magnesium and α-tocopheryl acetate (at 2 or 4 kg/Tn feed) on neutral lipid fraction of intramuscular fat.

	Treatments ^1^			SEM ^2^	*p* Value
	Control	MIX-2	MIX-4		
C14:0	1.46	1.40	1.44	0.051	0.7313
C16:0	24.73	24.79	24.58	0.359	0.9330
C16:1n9	0.18	0.19	0.19	0.010	0.5158
C16:1n7	3.86	3.68	3.69	0.193	0.7656
C17:0	0.19	0.20	0.17	0.008	0.1494
C17:1	0.19	0.20	0.19	0.010	0.6031
C18:0	12.81	13.11	12.96	0.359	0.4834
C18:1n9	46.30 ^a^	46.52 ^a^	44.65 ^b^	0.430	0.0092
C18:1n7	3.58	3.28	3.35	0.143	0.2576
C18:2n6	5.06 ^b^	5.01 ^b^	6.52 ^a^	0.207	0.0001
C18:3n3	0.27	0.26	0.24	0.012	0.2889
C20:0	0.24 ^a^	0.22 ^ab^	0.20 ^b^	0.009	0.0185
C20:1n9	0.82	0.81	0.77	0.036	0.7227
C20:3n6	0.06 ^b^	0.08 ^b^	0.11 ^a^	0.009	0.0006
C20:4n6	0.19 ^b^	0.21 ^b^	0.69 ^a^	0.015	0.0001
C20:5n3	0.02 ^a^	0.02 ^b^	0.01 ^b^	0.002	0.0001
C22:4	0.02 ^b^	0.01 ^b^	0.15 ^a^	0.005	0.0001
C22:5n3	0.02 ^a^	0.01 ^c^	0.01 ^b^	0.001	0.0001
C22:6n3	0.02 ^b^	0.01 ^b^	0.09 ^a^	0.004	0.0001
∑SAT ^3^	39.73	39.37	39.66	0.548	0.5999
∑MUFA ^4^	54.73 ^a^	54.88 ^a^	52.74 ^b^	0.477	0.0064
∑PUFA ^5^	5.54 ^b^	5.75 ^b^	7.60 ^a^	0.231	0.0001
∑n-6	5.22 ^b^	5.44 ^b^	7.26 ^a^	0.222	0.0001
∑n-3	0.32 ^ab^	0.32 ^b^	0.34 ^a^	0.014	0.0095
∑n-6/∑n-3	16.17 ^c^	17.37 ^b^	21.51 ^a^	0.576	0.0001

^1^ Control = Non additives; MIX-2 = 2 kg/Tn of oleuropein extract (600 mg/kg) with betaine (500 mg/kg), magnesium oxide (300 mg/kg) and α–tocopheryl acetate (200 mg/kg); MIX-4 = 4 kg/Tn of oleuropein extract (1200 mg/kg) with betaine (1000 mg/kg), magnesium oxide (600 mg/kg) and α–tocopheryl acetate (400 mg/kg); ^2^ SEM: Standard error of the mean, *n* = 10; ^3^ SAT: Sum of saturated fatty acids; ^4^ MUFA: Sum of monounsaturated fatty acids; ^5^ PUFA: Sum of polyunsaturated fatty acids. Different letters (^a–c^) indicate *p* < 0.05.

**Table 5:** Effect of dietary oleuropein extract combined with betaine, magnesium and α-tocopheryl acetate (at 2 or 4 kg/Tn feed) on free-fatty acid lipid fraction of intramuscular fat.

	Treatments ^1^			SEM ^2^	*p* Value
	Control	MIX-2	MIX-4		
C14:0	1.46	1.42	1.39	0.043	0.4104
C16:0	25.91 ^a^	25.23 ^ab^	23.88 ^b^	0.412	0.0034
C16:1n9	0.37 ^ab^	0.46 ^a^	0.35 ^b^	0.027	0.0159
C16:1n7	2.85	2.88	3.18	0.151	0.1896
C17:0	0.41	0.34	0.39	0.023	0.0912
C17:1	0.19 ^ab^	0.16 ^b^	0.21 ^a^	0.015	0.0095
C18:0	17.66	17.08	17.19	0.634	0.8020
C18:1n9	35.47	36.29	36.24	0.726	0.2736
C18:1n7	6.00	6.14	6.37	0.208	0.4128
C18:2n6	5.84	6.51	6.32	0.280	0.6219
C18:3n3	0.67 ^ab^	0.55 ^b^	0.71 ^a^	0.037	0.0127
C20:0	0.28 ^b^	0.24 ^b^	0.33 ^a^	0.011	0.0001
C20:1n9	0.75 ^ab^	0.70 ^b^	0.76 ^a^	0.035	0.0116
C20:3n6	0.14 ^b^	0.14 ^b^	0.23 ^a^	0.013	0.0001
C20:4n6	1.44	1.33	1.72	0.133	0.1629
C20:5n3	0.16	0.15	0.17	0.037	0.9809
C22:4n6	0.11 ^b^	0.13 ^b^	0.16 ^a^	0.013	0.0034
C22:5n3	0.08 ^b^	0.05 ^b^	0.11 ^a^	0.010	0.0003
C22:6n3	0.20 ^b^	0.19 ^b^	0.30 ^a^	0.021	0.0003
∑SAT ^3^	45.72	44.31	43.18	0.765	0.1166
∑MUFA ^4^	45.63	46.63	47.11	0.839	0.3019
∑PUFA ^5^	8.65 ^b^	9.06 ^ab^	9.72 ^a^	0.367	0.0654
∑n-6	1.69 ^b^	1.60 ^b^	2.11 ^a^	0.141	0.0339
∑n-3	0.58 ^b^	0.53 ^b^	0.81 ^a^	0.052	0.0009

^1^ Control = Non additives; MIX-2 = 2 kg/Tn of oleuropein extract (600 mg/kg) with betaine (500 mg/kg), magnesium oxide (300 mg/kg) and α–tocopheryl acetate (200 mg/kg); MIX-4 = 4 kg/Tn of oleuropein extract (1200 mg/kg) with betaine (1000 mg/kg), magnesium oxide (600 mg/kg) and α–tocopheryl acetate (400 mg/kg); ^2^ SEM: Standard error of the mean, *n* = 10; ^3^ SAT: Sum of saturated fatty acids; ^4^ MUFA: Sum of monounsaturated fatty acids; ^5^ PUFA: Sum of polyunsaturated fatty acids. Different letters (^a,b^) indicate *p* < 0.05.

**Table 6:** Effect of dietary oleuropein extract combination with betaine, magnesium and α-tocopheryl acetate (at 2 or 4 kg/Tn feed) on polar lipid fraction of intramuscular fat.

	Treatments ^1^			SEM ^2^	*p* Value
	Control	MIX-2	MIX-4		
C14:0	3.12	3.31	3.26	0.152	0.9216
C16:0	23.36 ^ab^	24.25 ^a^	23.07 ^b^	0.351	0.0245
C16:1n9	1.53 ^a^	1.62 ^a^	1.32 ^b^	0.051	0.0001
C16:1n7	0.73 ^b^	0.80 ^b^	1.07 ^a^	0.069	0.0002
C17:0	0.45 ^b^	0.45 ^b^	1.57 ^a^	0.114	0.0001
C17:1	0.28 ^b^	0.28 ^b^	1.22 ^a^	0.106	0.0001
C18:0	6.89	7.13	6.88	0.209	0.2522
C18:1n9	11.78	12.22	11.42	0.425	0.5524
C18:1n7	1.82 ^ab^	1.73 ^a^	1.83 ^a^	0.053	0.0549
C18:2n6	35.62 ^a^	32.66 ^b^	33.41 ^b^	0.503	0.0021
C18:3n3	0.38 ^a^	0.35 ^ab^	0.33 ^b^	0.010	0.0438
C20:0	0.07	0.06	0.07	0.005	0.4129
C20:1n9	0.17	0.17	0.16	0.008	0.1199
C20:3n6	1.06 ^ab^	1.10 ^a^	1.04 ^b^	0.028	0.0583
C20:4n6	9.44	10.30	9.99	0.272	0.5264
C20:5n3	0.25	0.26	0.25	0.009	0.9186
C22:4n6	1.72	1.84	1.62	0.064	0.3686
C22:5n3	1.04	1.13	1.11	0.033	0.6494
C22:6n3	0.29	0.34	0.39	0.030	0.691
∑SAT ^3^	33.90 ^b^	35.20 ^a^	34.84 ^ab^	0.335	0.0517
∑MUFA ^4^	16.31 ^b^	16.82 ^ab^	17.01 ^a^	0.463	0.0563
∑PUFA ^5^	49.50 ^a^	47.64 ^b^	47.76 ^b^	0.515	0.0074
∑n-6	12.50	13.58	13.04	0.297	0.3222
∑n-3	1.58	1.73	1.74	0.057	0.6231
∑n-6/∑n-3	7.96	7.89	7.56	0.187	0.6089

^1^ Control = Non additives; MIX-2 = 2 kg/Tn of oleuropein extract (600 mg/kg) with betaine (500 mg/kg), magnesium oxide (300 mg/kg) and α–tocopheryl acetate (200 mg/kg); MIX-4 = 4 kg/Tn of oleuropein extract (1200 mg/kg) with betaine (1000 mg/kg), magnesium oxide (600 mg/kg) and α–tocopheryl acetate (400 mg/kg); ^2^ SEM: Standard error of the mean, *n* = 10; ^3^ SAT: Sum of saturated fatty acids; ^4^ MUFA: Sum of monounsaturated fatty acids; ^5^ PUFA: Sum of polyunsaturated fatty acids. Different letters (^a,b^) indicate *p* < 0.05.

## Data Availability

Not Applicable.
